# ‘Imposter participants’ in online qualitative research, a new and increasing threat to data integrity?

**DOI:** 10.1111/hex.13724

**Published:** 2023-02-16

**Authors:** Damien Ridge, Laurna Bullock, Hilary Causer, Tamsin Fisher, Samantha Hider, Tom Kingstone, Lauren Gray, Ruth Riley, Nina Smyth, Victoria Silverwood, Johanna Spiers, Jane Southam

**Affiliations:** ^1^ School of Social Sciences, College of Liberal Arts & Sciences University of Westminster London UK; ^2^ School of Medicine Keele University Newcastle under Lyme & Staffordshire UK; ^3^ School of Health Sciences University of Surrey Guildford UK; ^4^ Haywood Rheumatology Centre Midlands Partnership NHS Foundation Trust Stoke‐on‐Trent UK; ^5^ Research and Innovation Midlands Partnership NHS Foundation Trust Stafford UK

To the Editor,

We would like to share the experiences we have recently had while conducting online qualitative studies. We believe people have contacted members of our research teams, offering to participate in interviews for a health study, who were not the people they initially purported to be. We reimburse participants to cover time and expenses for their involvement in research, with varying views on the complex issue of incentivization.^1^ We also acknowledge the benefits of adapting to COVID‐19 via online qualitative research: this data can be as rich and valuable as that obtained in face‐to‐face settings^2^; social media can provide excellent avenues of recruitment (especially for seldom heard participants); while data collection can be more economical in terms of time and cost, including compared to physical R&D site negotiations. Researchers collecting data in this way can more easily reach large geographical areas, and online is also a greener option than travelling long distances for interviews.^3^, ^4^ Additionally, it is not unheard of for participants to falsify their identities in face‐to‐face research.^5^

Nevertheless, three recent online qualitative studies in which we recruited healthcare professionals, and a further two online studies (recruiting older adults and those with a long‐term health condition for interview) aroused suspicion amongst our research teams regarding the authenticity of some potential participants. It took research teams varying amounts of time to piece together evidence of the inauthenticity in participants/interviews, especially in cases where participants gave apparently plausible accounts and/or were difficult to understand. On reflection, the following indicators suggested to us that these may have been ‘imposter’ participants:

(1) Suspect participants sometimes cited recruitment sites that were not used by the study
(2) More than one suspect participant gave the same social media platform as their source for learning about the study, despite the use of multiple platforms for recruitment in a study
(3) Participants responded quickly, sometimes within minutes, of recruitment advertisements being posted on social media, and some of these posted multiple follow‐up emails in quick succession
(4) Emails from multiple suspect participants had similar configurations and frequently came from the same email platform
(5) Blank subject lines in emails expressing interest in the study (as well as emails that were only a sentence or two long) were common, with some potential participants copying text from recruitment ads verbatim
(6) In some cases, a single participant was suspected of slightly changing their story and sociodemographics to complete multiple interviews, which could be difficult to detect quickly, especially where more than one researcher was conducting interviews (i.e., suspect interviews were spread across different data collectors)
(7) Researchers noted matches between participants' voices, stories and limitations in accounts of the health condition under investigation, as well as similar sociodemographic profiles
(8) Concerns about timing and methods of payment were a priority for the participant
(9) There were requests for payment via PayPal or in the form of vouchers, and a reticence to share bank details for bankers' automated clearing system (BACS) payments, citing concerns around confidentiality
(10) Researcher requests for details of participants' professional backgrounds resulted in participants verbatim copying a profession from the examples listed in the recruitment advertisement
(11) ‘Health professional’ participants declined to give their work (e.g., National Health Service [NHS]) email for verification purposes, claiming concerns around anonymity (even if conversations about the study were permitted to be carried out via personal email)
(12) Requests for an institutional email address to authenticate participants' profession before payment resulted in no further communication from those participants
(13) Suspect participants who were interviewed via video links chose to keep their cameras off, citing a range of reasons, for example, shyness
(14) Preference was expressed for online interviews (albeit with the camera turned off) over telephone interviews, and there was a reticence to disclose a telephone contact number
(15) Interview questions elicited vague answers, and participants seemed distracted or otherwise occupied during interviews

This phenomenon is not something we, as a group of researchers, had come across pre‐COVID. We wondered if it might be related to the accelerated move to online research methods necessitated by the pandemic,^6^ and especially the cost of living crisis in the United Kingdom and globally,^7^ with people needing to find ways of boosting their income in the face of soaring inflation. Nevertheless, we expect that motivations varied.

Importantly, this is not a new problem; Hydock^8^ discovered that a ‘small but nontrivial portion of participants’ engaging in online survey studies ‘misrepresented their identity for the chance of financial gain’ (p. 1566). Other authors have suggested that the falsification of identity in online surveys is more widespread than suggested by Hydock.^9^, ^10^ Roehl and Harding^11^ argue that participants in online qualitative research have a good opportunity to perform an identity to volunteer for research studies when they do not meet inclusion criteria. They (and those discussing self‐reported survey research) recommend that researchers plan their recruitment procedures, data collection and approach to analyses to protect data from ‘dishonest’ participants.^8^, ^12^ We note that fraudulent participation in qualitative research is an apparently newer and potentially more complex endeavour than carrying out online survey response scams.

How can we better mitigate against fraud? For studies involving NHS staff, researchers took the approach of asking potential participants to verify their identities via NHS email, while allowing conversations about the study to happen via personal emails. They also concluded that it is better to prioritize online video platforms, with the camera switched on, than use the telephone for interviews where possible, whilst being aware that telephone options are still important for accessibility for many participants. Others among us asked that participants turn their video on at least for a preinterview chat, or at the start of the interview, to discourage imposter participants. Further steps taken by researchers to deal with potential imposters included:

(1) Considering closing recruitment from the social media source singled out by suspect participants
(2) Participants sending subsequent suspect requests to participate politely advised that recruitment had closed for their particular sociodemographic
(3) Checking email configurations and formats of all new participation requests for patterns
(4) Being up‐to‐date with information security as well as means for identifying phishing (and accessing training available) in researchers' organizations
(5) Checking names on professional registers to authenticate professional participants
(6) Considering BACS payments for reimbursement, as opposed to vouchers
(7) Where participants give similar or odd stories, researchers review these particular accounts
(8) Where multiple study team members are carrying out qualitative interviews, it is helpful for interviewers to meet and regularly review accounts in the data, thus ensuring any potential issue is identified as quickly as possible
(9) Removal of the suspect interview data from the study

Lawlor et al.^10^ posited that, in online survey research, it is important to have a systematic process for determining the level of suspicion required to remove potentially unreliable data. Researchers conducting qualitative studies should also carefully plan for this possibility by, for example, writing an ‘online imposter participant protocol’ to ensure trustworthy data. Discussions about a protocol should be part of the planning stage for each study, and continue as circumstances arise during recruitment or data collection. We suggest the following questions and approaches that researchers may use to guide this process:

1. During recruitment, how could I verify that the participants meet the inclusion criteria? How confident can I be in this information?
2. During data collection, was the participant hesitant or flustered when asked probing questions for additional detail?
3. Did I document impressions of honesty in my reflexive journal?
4. Did the participant's answers make sense, and were they detailed enough about the topic being researched?

Jones et al.^13^ advise that qualitative data is rich in detail, making it easier to spot dubious data. However, just because a participant does not provide a detailed description, does not necessarily mean they are an imposter. Not all qualitative interviews go well or result in illuminating data. Balancing a need to build rapport with participants, while being cognizant of potential imposters, is a first step for researchers to determine whether a participant is indeed an imposter or is simply giving an interview that yields less substantial  data.

In conclusion, recruiting participants online and conducting virtual interviews are likely to become increasingly popular practices for qualitative researchers. All qualitative researchers using online methods should consider and prepare a protocol for dealing with potential imposter participants in their qualitative studies. And at a time when even recent internet‐mediated research ethics guidelines are yet to get to grips with the problem,^14^ ethics committees should be seen as having an important role in scrutinizing online ‘imposter participant protocols’. For example, interviewers can feel uneasy about doubting the authenticity of participants, meaning there are impacts on the researcher. We believe it is important for principal investigators to permit researchers to voice uncertainty about authenticity in research teams, and to debrief with members in cases of suspected imposter participants, thus addressing issues like self‐blame. Additionally, discussing this potential problem in the planning stages of studies with patient and public involvement or advisory groups is likely to prove valuable in helping us to get the balance right in giving voice to our participants and maintaining the integrity of our research.

Should we consider any other factors? For example, if you become aware that a participant is being dishonest during an interview, how do you handle this in real time? What kinds of safety risks might a researcher be opening themselves up to from an imposter participant? We would welcome discussion amongst readers of HEX on the issue of imposter participants in qualitative research.

## AUTHOR CONTRIBUTIONS

**Damien Ridge**: Co‐ordination of letter drafting; conceptualization; contribution of content; review and editing; and approval of the final manuscript. **Laurna Bullock**: Conceptualization; review and editing; and approval of the final manuscript. **Hilary Causer** and **Samantha Hider**: Conceptualization; contribution of content; review and editing; and approval of the final manuscript.   **Tom Kingstone**: Conceptualization; contribution of content; supported drafting of the letter; review and editing; and approval of the final manuscript. **Lauren Gray**: Review and editing; and approval of the final manuscript. **Ruth Riley**, **Tamsin Fisher** and **Victoria Silverwood**: Conceptualisation; review and approval of the final manuscript. **Nina Smyth**: Contribution of content; conceptualization; supported drafting of the letter; review and editing; and approval of the final manuscript. **Johanna Spiers**: Contribution of content; review and editing; copy editing and approval of the final manuscript.   **Jane Southam**: Review and approval of the final manuscript.

## CONFLICT OF INTEREST STATEMENT

The authors of this letter work closely with Professor Carolyn Chew‐Graham, who is Editor in Chief of *Health Expectations*.

## ETHICS STATEMENT

There are no ethical requirements for this letter/opinion piece.

## Data Availability

Data sharing is not applicable as no new data is generated.

## References

[1] Gelinas L , Largent EA , Cohen IG , Kornetsky S , Bierer BE , Fernandez Lynch H . A framework for ethical payment to research participants. N Engl J Med. 2018;378:766‐771. 29466147 10.1056/NEJMsb1710591

[2] Archibald MM , Ambagtsheer RC , Casey MG , Lawless M . Using Zoom videoconferencing for qualitative data collection: perceptions and experiences of researchers and participants. Int J Qual Methods. 2019;18:160940691987459. 10.1177/1609406919874596

[3] Kobakhidze MN , Hui J , Chui J , González A . Research disruptions, new opportunities: re‐imagining qualitative interview study during the COVID‐19 pandemic. Int J Qual Methods. 2021;20:160940692110515. 10.1177/16094069211051576

[4] Whitaker C , Stevelink S , Fear N . The use of Facebook in recruiting participants for health research purposes: a systematic review. J Med Internet Res. 2017;19(8):e290. 10.2196/jmir.7071 28851679 PMC5594255

[5] Flicker S . “Ask me no secrets, I'll tell you no lies”: what happens when a respondent's story makes no sense. Qual Rep. 2004;9(3):528‐537.

[6] Saarijärvi M , Bratt E‐L . When face‐to‐face interviews are not possible: tips and tricks for video, telephone, online chat, and email interviews in qualitative research. Eur J Cardiovasc Nurs. 2021;20(4):392‐396. 10.1093/eurjcn/zvab038 33893797 PMC8135391

[7] Hall SG , Tavlas GS , Wang Y . Drivers and spillover effects of inflation: the United States, the euro area, and the United Kingdom. J Int Money Finance. 2023;131:102776. 10.1016/j.jimonfin.2022.102776

[8] Hydock C . Assessing and overcoming participant dishonesty in online data collection. Behav Res Methods. 2018;50(4):1563‐1567. 29274007 10.3758/s13428-017-0984-5

[9] Pozzar R , Hammer MJ , Underhill‐Blazey M , et al. Threats of bots and other bad actors to data quality following research participant recruitment through social media: cross‐sectional questionnaire. J Med Internet Res. 2020;22(10):e23021. 10.2196/23021 33026360 PMC7578815

[10] Lawlor J , Thomas C , Guhin AT , Kenyon K , Lerner MD , Drahota A . Suspicious and fraudulent online survey participation: introducing the REAL framework. Methodol Innov. 2021;14(3):205979912110504. 10.1177/20597991211050467

[11] Roehl JM , Harland DJ . Imposter participants: overcoming methodological challenges related to balancing participant privacy with data quality when using online recruitment and data collection. Qual Rep. 2022;27(11):2469‐2485.

[12] Chandler JJ , Paolacci G . Lie for a dime: when most prescreening responses are honest but most study participants are impostors. Soc Psychol Pers Sci. 2017;8(5):500‐508. 10.1177/1948550617698203

[13] Jones A , Caes L , Rugg T , Noel M , Bateman S , Jordan A . Challenging issues of integrity and identity of participants in non‐synchronous online qualitative methods. Methods Psychol. 2021;5:100072. 10.1016/j.metip.2021.100072

[14] British Psychological Society . Ethics Guidelines for Internet‐Mediated Research. British Psychological Society; 2021.

